# Motion Compensation of Moving Targets for High Range Resolution Stepped-Frequency Radar

**DOI:** 10.3390/s8053429

**Published:** 2008-05-23

**Authors:** Yimin Liu, Huadong Meng, Hao Zhang, Xiqin Wang

**Affiliations:** Department of Electronic Engineering, Tsinghua University, Beijing 100084, PR China

**Keywords:** high resolution, radar imaging, stepped-frequency, motion compensation

## Abstract

High range resolution (HRR) profiling using stepped-frequency pulse trains suffers from range shift and the attenuation/dispersion of range profiles while the target of interest is moving. To overcome these two drawbacks, a new algorithm based on the maximum likelihood (ML) estimation is proposed in this paper. Without altering the conventional stepped-frequency waveform, this algorithm can estimate the target velocity and thereby compensate the phase errors caused by the target's motion. It is shown that the velocity can be accurately estimated and the range profile can be correctly reconstructed.

## 1. Introduction

High resolution radar is an area of vigorous research and development in recent years. It is known that radar's range resolution is inversely proportional to its bandwidth. Therefore, the increase in bandwidth correspondingly improves the radar's range resolution. However, the wideband radar pulses complicate the design of transmitters and receivers. Also, such radar receivers are subject to potential interference from other sources.

To overcome these drawbacks, Ruttenberg [1] introduced a kind of radar waveforms containing a train of pulses whose carrier frequencies were different from each other. In 1984, the stepped-frequency pulse trains, with the carrier frequency of each pulse shifting linearly at a constant step, were introduced by Einstein, T.H. [2]. The total bandwidth of the pulse train can be synthesized together and the high range resolution profile (HRRP) can be generated by the inverse discrete Fourier transform (IDFT). Since then, the stepped-frequency pulse trains have been widely used in high range resolution (HRR) radars.

As mentioned in [2–3], the stepped-frequency radars suffer from distortion arising from the target's motion. An uncompensated non-zero radial velocity is found to have two effects on the HRRP. The first one is the circular shift in the HRRP. The second effect is the attenuation and dispersion of the HRRP [2]. These effects degrade the performance of stepped-frequency radars. Recently, several investigations have been proposed to mitigate the distortion caused by target's motion. In [4], two effective methods called reverse-count method and amplitude-interpolating method were introduced to solve the range shift effect. However, the attenuation/dispersion effect was not taken into account. In [5], the distortion of the HRRP from a perturbed target was analyzed, and a numerical model was developed to simulate the distortion effects. A kind of phase-coded, stepped-frequency waveforms, which had lower Doppler sensitivity than the traditional ones, was introduced by Temple in [6]. In [7], two successive stepped-frequency pulse trains were used to eliminate the phase errors of the moving target. Li et al. [8] introduced a new method using multiple stepped-frequency pulse trains and the robust phase unwrapping theorem to estimate the range and the velocity of the target. However, the majority of these methods [6–8] focused on the technique trends, which needed transmitting and receiving new kinds of waveforms. As a result, their implementations on those traditional in-service stepped-frequency radars were restrained.

A new motion compensation algorithm, based on the maximum likelihood (ML) estimation, is provided in this paper. It will be shown that this algorithm can estimate the target's radial velocity accurately and reconstruct the distorted HRRP successfully. Without altering the conventional waveforms, the new algorithm can be implemented on the in-service stepped-frequency radars.

The remainder of this paper is organized as follows. In Section 2, the signal model of moving targets in stepped-frequency radar systems is formulated. In Section 3, the ML estimator of the radial velocity is derived. Then, using the fast Fourier transforms to reduce the computational load, the new algorithm is proposed. In Section 4, some numerical examples are given to demonstrate the performance of the proposed algorithm. Section 6 presents the conclusions drawn from this work.

## 2. Signal Model

A stepped-frequency pulse train is a series of pulses modulated with different carrier frequencies. The carrier frequency of the first pulse is *f_c_* and those of the rest *N*−1 subsequent pulses are *f_c_*+*n*Δ*f*, *n*=1,2,…,*N*−1, where *N* is the number of pulses and Δ*f* is the frequency step size. Then, the *n*th transmitted pulse is

(1) $$s_{n}\left(t\right)=\text{rect}\left(\frac{t-nT_{r}}{T}\right)\cdot e^{j2\pi \left(f_{c}+n\Delta f\right)t},n=0,1,\ldots ,N-1,$$

where rect(·) is the rectangular function, *T* is the pulse width, *T_r_* is the pulse repetition interval (PRI) and the amplitude of the transmitted pulse is supposed to be 1. Supposing that the target is an ideal point-like scatterer, and disregarding both the energy divergence on wave propagation paths and the variation of target's reflection factor, the *n*th received pulse is

(2) $$r_{n}\left(t\right)=\alpha \cdot \text{rect}\left(\frac{t-2\left(R_{0}+V_{R}nT_{r}\right)/c-nT_{r}}{T}\right)\cdot e^{j2\pi \left(f_{c}+n\Delta f\right)\left[t-2\left(R_{0}+V_{R}nT_{r}\right)/c\right]}+w_{n}\left(t\right),$$

where *α* is the amplitude of the received signal, *c* is the speed of light, *w_n_*(*t*) is the additive noise, *R*_0_ and *V_R_* are the initial range and radial velocity of the target. As in most studies on the stepped-frequency waveforms, it is assumed that *V_R_* is constant in the whole coherent processing interval (CPI), and the target range migration does not exceed *cT*/2, which is the rough resolution determined by a single pulse. Each pulse is demodulated with its corresponding carrier frequency, thus the baseband signal of the *n*th pulse is

$$x_{n}\left(t\right)=\alpha \cdot \text{rect}\left(\frac{t-2\left(R_{0}+V_{R}nT_{r}\right)/c-nT_{r}}{T}\right)\cdot e^{-j4\pi \left(f_{c}+n\Delta f\right)\left(R_{0}+V_{R}nT_{r}\right)/c}+w_{n}\left(t\right).$$

The sample of the received baseband signal is

(3) $$x_{n}=\alpha \cdot e^{-j4\pi \left(R_{0}f_{c}+R_{0}\Delta fn\right)/c}\cdot e^{-j4\pi \left(f_{c}V_{R}T_{r}n+V_{R}T_{r}\Delta fn^{2}\right)/c}+w_{n}.$$

The first phase term of the right side of (3) is caused by the initial range of the target, and the second phase term is the additional phase term caused by the radial velocity. When the target and the radar are relatively stationary, i.e., *V_R_*=0, one can use the IDFT to estimate the target's range and to generate the HRRP. However, if the target and the radar are not relatively stationary, as mentioned in [2–3], the linear component of the additional phase term will cause the circular range shift and the quadratic component will cause the attenuation/dispersion of the range profiles. Both of them are significant distortions in using stepped-frequency pulse trains to generate the HRRP. However, once the *Vr* is able to be estimated, the additional phase term can be compensated, and the HRRP can be reconstructed as well.

Denoting

(4) $$\eta =-4\pi \left(R_{0}\Delta f+f_{c}V_{R}T_{r}\right)/c,$$

and

(5) $$\mu =-4\pi V_{R}T_{r}\Delta f/c,$$

(3) can be re-written as

(6) $$x_{n}=\alpha \cdot e^{j\left(\eta n+\mu n^{2}\right)}+w_{n},$$

Supposing that a target is composed by *K* scatterers and the amplitude of the received pulse from each scatterer is *α_k_*, *k* = 0,1,…,*K* − 1, the baseband signal is

(7) $$g_{n}=\sum_{k=0}^{K-1}\alpha_{k}\cdot e^{j\left(\eta_{k}n+\mu_{k}n^{2}\right)}+w_{n},$$

where *η_k_* and *μ_k_* are determined by the range and velocity of each scatterer as proposed in (4) and (5). Denoting Ω*_k_*= [1, *e^j^*^(^*^ηk^*^1+^*^μk^*^12)^, *e^j^*^(^*^ηk^*^2+^*^μk^*^22)^,…,*e^j^*^(^*^ηk^*^(^*^N^*^−1)+^*^μk^*^(^*^N^*^−1)2)^]*^T^*, where □*^T^* is the transpose operation, Equation (7) can be re-written in the matrix form, as

(8) g=Ωα+w,

where **g** = [*g*_0_, *g*_1_,…, *g_N_*_−1_], ***w*** = [*w*_0_, *w*_1_,…, *w_N_*_−1_], **α** = [*α*_0_, *α*_1_,…,*α_K_*_−1_]*^T^*, and **Ω** = [Ω_0_, Ω_1_,…, Ω*_K_*_−1_].

## 3. Velocity Estimation and Motion Compensation

### 3.1. Maximum Likelihood Estimation of the Target Velocity

The motion compensation and range profiling of a moving target can be seen as the estimation of the scatterers' amplitudes, ranges, and velocities. According to (7), this problem is the same as the estimation of ${\left\{\alpha_{k},\eta_{k},\mu_{k}\right\}}_{k=0}^{K-1}$ through the observations vector **g**. Supposing that the noise is additive white Gaussian noise (AWGN), the ML estimation of ${\left\{\alpha_{k},\eta_{k},\mu_{k}\right\}}_{k=0}^{K-1}$ can be obtained by minimizing the following cost function:

(9) $$C\left({\left\{\alpha_{k},\eta_{k},\mu_{k}\right\}}_{k=0}^{K-1}\right)={\left\|\text{g}-\Omega \alpha \right\|}^{2}.$$

where ‖□‖ denotes the Euclidean norm. Minimizing (9; with respect to **α** yields

(10) $$\hat{\alpha }={\left(\Omega^{H}\Omega \right)}^{-1}\Omega^{H}\text{g},$$

where □*^H^* is the conjugate transpose operation. Substituting (10) into (9), we get

(11) $$\begin{array}{ll} C\left({\left\{\alpha_{k},\eta_{k},\mu_{k}\right\}}_{k=0}^{K-1}\right) & ={\left\|g-\Omega \hat{\alpha }\right\|}^{2}\\ & =g^{H}g-g^{H}\Omega {\left(\Omega^{H}\Omega \right)}^{-1}\Omega^{H}g. \end{array}$$

Then, the ML estimation of ${\left\{\eta_{k},\mu_{k}\right\}}_{k=0}^{K-1}$ can be achieved through

(12) $${\left\{{\hat{\eta }}_{k},{\hat{\mu }}_{k}\right\}}_{k=0}^{K-1}=\underset{\eta_{k},\mu_{k}}{\arg\max}\left\{g^{H}\Omega {\left(\Omega^{H}\Omega \right)}^{-1}\Omega^{H}g\right\}.$$

As assumed in the previous section, the radial velocity of each scatterer is the same. Thus, *μ*_0_ = *μ*_1_ = … = *μ_K_*_−1_ = *μ*, and

(13) $$\Omega_{k}=\text{diag}\left\{1,e^{j\mu 1^{2}},e^{j\mu 2^{2}},\ldots ,e^{j\mu {\left(N-1\right)}^{2}}\right\}{\left[1,e^{j\left(\eta_{k}1\right)},e^{j\left(\eta_{k}2\right)},\ldots ,e^{j\left(\eta_{k}\left(N-1\right)\right)}\right]}^{T},k=0,\ldots ,K-1.$$

Supposing that the number of pulses *N*□ 1 and the range intervals between the scatterers are larger than Δ*R* (the range resolution, $\Delta R=\frac{c}{2N\Delta f}$ [3]), we have

(14) $${\left(\Omega^{H}\Omega \right)}^{-1}\approx \frac{1}{N}I,$$

where **I** is the identity matrix. Substitute (14) into (12), and then the ML estimation of ${\left\{\eta_{k},\mu \right\}}_{k=0}^{K-1}$ can be modified as

(15) $$\begin{array}{ll} {\left\{{\hat{\eta }}_{k},\hat{\mu }\right\}}_{k=0}^{K-1} & =\underset{\eta_{k},\mu }{arg max}\left\{{\text{g}}^{H}\Omega \Omega^{H}g\right\}\\ & =\underset{\eta_{k},\mu }{arg max}\left\{\sum_{k=0}^{K-1}{\left\|g^{H}\text{diag}\left\{1,e^{j\mu 1^{2}},e^{j\mu 2^{2}},\ldots ,e^{j\mu {\left(N-1\right)}^{2}}\right\}{\left[1,e^{j\left(\eta_{k}1\right)},e^{j\left(\eta_{k}2\right)},\ldots ,e^{j\left(\eta_{k}\left(N-1\right)\right)}\right]}^{T}\right\|}^{2}\right\}. \end{array}$$

According to (5), the velocity of the target can be achieved as

(16) $${\hat{V}}_{R}=-\hat{\mu }c/\left(4\pi T_{r}\Delta f\right).$$

So the quadratic phase term caused by radial velocity can be compensated through

(17) $$\text{g}\prime =\text{diag}\left\{1,e^{-j\hat{\mu }1^{2}},e^{-j\hat{\mu }2^{2}},\ldots ,e^{-j\hat{\mu }{\left(N-1\right)}^{2}}\right\}g.$$

And the total additional phase term caused by radial velocity can be compensated, as

(18) $$x_{n}\prime =x_{n}e^{j4\pi {\hat{V}}_{R}\left(f_{c}T_{r}n+T_{r}\Delta fn^{2}\right)/c}.$$

Finally, the HRRP of the moving target can be generated by the IDFT of [*x*_0_′, *x*_1_′,…, *x_N_*_−1_′].

### 3.2. The Motion Compensation and Profile Reconstruction Algorithm

With the above-mentioned preparation, the motion compensation algorithm will be introduced in this section. If the number of scatterers *K* is supposed to be known, the ML estimation of the parameters are obtained as follows.

Firstly, the parameter *μ* is assumed to be known, as *μ*=*μ′*. Then, the additional quadratic phase term caused by radial velocity is compensated by using (17).

Secondly, the FFT is adopted to estimate the ${\left\{\eta_{k}\right\}}_{k=0}^{K-1}$ in (15) or the purpose o reducing the computation load. The FFT is performed on the compensated signal vector **g**′, and the squares of amplitudes of the FFT results are calculated, as **F***_μ_*_′_ = ‖FFT(**g**′)‖^2^. The largest *K* components of vector **F***_μ_*_′_ are chosen, and their sum is denoted as *S_μ_*_′_. The *S_μ_*_′_ is a function of *μ*′. The estimation of ${\left\{\eta_{k}\right\}}_{k=0}^{K-1}$ is the frequency corresponding to these largest *K* components.

Thirdly, the *S_μ_*_′_ is maximized with respect to *μ*′. Then, the ML estimation of *μ*, denoted as *μ̂*, can be obtained through

(19) $$\hat{\mu }=\underset{\mu \prime \in [-4\pi V_{R\min}T_{r}\Delta f/c,-4\pi V_{R\max}T_{r}\Delta f/c]}{\arg\max}\left\{S_{\mu \prime }\right\},$$

where [*V_R_*_min_, *V_R_*_max_] is the search space of the target radial velocity.

Finally, the estimation of target radial velocity *V̂_R_* can be derived according to (16). Then, applying the first and second step given above, the ${\left\{{\hat{\eta }}_{k}\right\}}_{k=0}^{K-1}$ can be achieved. The **α̂** is finally obtained through (10).

As the FFT is not exactly equivalent to the ML estimator of ${\left\{\eta_{k}\right\}}_{k=0}^{K-1}$ in the second step, we call the above procedure the simplified ML estimation (SMLE). As long as the estimation of radial velocity is obtained, (18) can be used to compensate the motion effect. The IDFT can be performed on the compensated signal to generate the undistorted HRRP.

However, if the target is non-cooperative, the number of scatterers *K* cannot be supposed as a prior. Thus, one should estimate *K* before applying the SMLE algorithm proposed here. The minimum description length (MDL) criterion introduced by Schwarz [9] is adopted here to estimate the number of scatterers. Schwarz's approach is based on Bayesian arguments. It is assumed that each hypothesis of *K* can be assigned a prior probability, and proposed to select the *K* that yields the maximum posterior probability. Using the MDL criterion, the number of scatterers *K* can be obtained by

(20) $$\underset{K}{\arg\min}\left\{\text{MDL}\left(K\right)=-\log M_{k}\left[x_{0},x_{1},\ldots ,x_{N-1}|{\left\{{\hat{\alpha }}_{k},{\hat{\eta }}_{k},{\hat{\mu }}_{k}\right\}}_{k=0}^{K-1}\right]+\frac{1}{2}K\log N\right\},$$

where ${\left\{{\hat{\alpha }}_{k},{\hat{\eta }}_{k},{\hat{\mu }}_{k}\right\}}_{k=0}^{K-1}$ is the ML estimate o the parameters, and $\log M_{k}\left[x_{0},x_{1},\ldots ,x_{N-1}|{\left\{{\hat{\alpha }}_{k},{\hat{\eta }}_{k},{\hat{\mu }}_{k}\right\}}_{k=0}^{K-1}\right]$ is log-likelihood of the ML estimate of the parameters.

An example is introduced here to validate the MDL criterion in our topic. We consider a moving target including four scatterers. The number of pulses in the stepped-frequency train is 512, and the signal-to-noise ratio (SNR) is 0 dB. The SMLE algorithm is used to yield the ML estimation of parameters and the following values for the MDL criterion (see Table 1).

The minimum of the MDL is obtained, as expected, for the *K* = 4.

With the MDL criterion and the SMLE algorithm proposed above, the scatterer number and the velocity of the target of interest can be estimated iteratively as follows.

Step 1 Assume that the number of scatterers is *K*=1.
Step 2 Obtain scatterers' parameters by using SMLE.
Step 3 Calculate the MDL(*K*).
Step 4 Assume *K*=*K*+1, and repeat *Step 2* and *Step 3*.

*Remaining Steps* Continue the similar steps, until the minimum MDL(*K*) is achieved. Then, use (16) and (18) to compensate for the radial velocity, and perform the IDFT on the compensated signal to generate the reconstructed HRRP.

## 4. Numerical Examples

In this section, some numerical examples are given to show the performance of the proposed new algorithm. The noise is AWGN in all of these simulations, and the parameters of the radar waveform used in this section are shown in Table 2.

**Figure 1.**
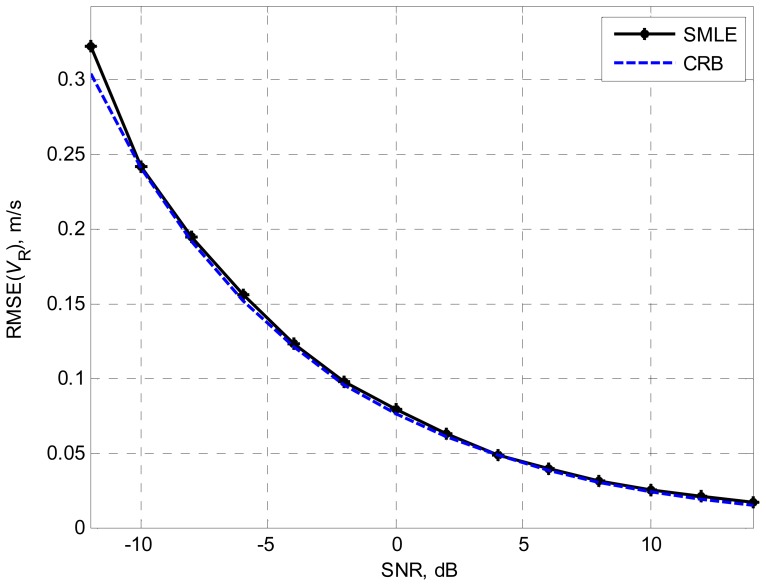
Root mean square error of the target velocity versus SNR.

First, we consider a moving target including only one scatterer. The range and the velocity of the target are 128Δ*R* and 100m/s, respectively. The number of scatterers *K* is supposed to be known in this simulation and the SMLE algorithm is used to estimate the velocity of the target. 2000 Monte–Carlo trials are run. The results are presented in comparison with the Cramer–Rao bound (CRB) [10–11]. The accuracy of the estimated velocity is measured by the root mean square error (RMSE), defined as $\sqrt{\frac{1}{N_{r}}\sum_{i=1}^{N_{r}}{\left({\hat{V}}_{R}-V_{R}\right)}^{2}}$, where *N_r_*=2000. Figure 1 shows the simulation results of the SMLE for target velocity versus various SNR. For SNR of −10dB or higher, the SMLE algorithm reaches the optimum estimation accuracy.

Another moving target including seven scatterers is considered. The ranges of the scatterers are 140Δ*R*, 153Δ*R*, 186Δ*R*, 195Δ*R*, 208Δ*R*, 251Δ*R*, and 258Δ*R*. Their amplitudes are 0.2, 0.3, 0.5, 0.7, 1.0, 0.7, and 0.2, respectively. And the target radial velocity is 100m/s.

**Figure 2.**
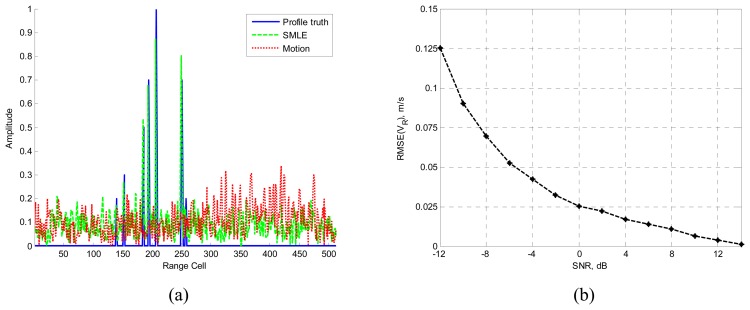
Simulation results of the moving target including seven scatterers.

The number of scatterers is obtained through the MDL criterion and the radial velocity is estimated by the SMLE. Simulation results are presented in Figure 2.

Figure 2(a) is the comparison between profile truth, profiling result with motion compensation and without motion compensation. The SNR is −5dB. It is shown that the profile result without motion compensation (the red dotted line) is badly attenuated and distorted, when compared with the profile truth with no motion (the blue solid line). However, the target velocity can be estimated through the algorithm proposed in this paper, and the HRRP is correctly reconstructed (the green dashed line). Figure 2(b) shows the accuracy of the estimated velocity.

## 5. Conclusion

In this paper, a new algorithm based on the ML estimation is proposed for HRR profiling of moving targets. This algorithm can be implemented on the in-service stepped-frequency radar systems, without changing their waveforms and system structures. The performance of this algorithm is guaranteed by the asymptotical optimality of the ML estimation [12]. The simulation results also show that the new algorithm can estimate the target velocity accurately and then reconstruct the HRRP correctly. Future work could include investigations into fast algorithms for the SMLE and how to extend this algorithm for maneuvering targets.

## Figures and Tables

**Table 1.** An example of the MDL criterion.

*K*	1	2	3	4	5	6	7	8	9	10
MDL	1100	924.99	748.11	572.3	574.9	576.12	577.36	578.7	580.14	581.6

**Table 2.** Parameters of the simulated stepped-frequency pulse train.

***Parameter***	***Value***
Radar center frequency (*f_c_*)	9 GHz
Frequency step size (Δ*f*)	1 MHz
Pulse number (*N*)	512
Range resolution (Δ*R*)	0.293 m
Pulse repetition interval (PRI)	1 ms
Pulse width (*T*)	0.5 μs
